# Succinate Dehydrogenase and Ribonucleic Acid Networks in Cancer and Other Diseases

**DOI:** 10.3390/cancers12113237

**Published:** 2020-11-03

**Authors:** Cerena Moreno, Ruben Mercado Santos, Robert Burns, Wen Cai Zhang

**Affiliations:** Department of Cancer Division, Burnett School of Biomedical Sciences, College of Medicine, University of Central Florida, 6900 Lake Nona Blvd, Orlando, FL 32827, USA; cerenamoreno@Knights.ucf.edu (C.M.); ruben.mercado30@Knights.ucf.edu (R.M.S.); robertburns@knights.ucf.edu (R.B.)

**Keywords:** succinate dehydrogenase, cancer, disease, tricarboxylic acid cycle, electron transport chain, metabolism, reactive oxygen species, non-coding RNA, RNA-editing, RNA-modification

## Abstract

**Simple Summary:**

Although the dysfunction of the succinate dehydrogenase complex in mitochondria leads to cancer and other diseases due to aberrant metabolic reactions and signaling pathways, it is not well known how the succinate dehydrogenase complex is regulated. Our review highlights that non-coding ribonucleic acids (RNAs), RNA editing enzymes, and RNA modifying enzymes regulate expressions and functions of the succinate dehydrogenase complex. This research will provide new strategies for treating succinate dehydrogenase-relevant diseases in a clinic.

**Abstract:**

Succinate dehydrogenase (SDH) complex connects both the tricarboxylic acid (TCA) cycle and the electron transport chain (ETC) in the mitochondria. However, *SDH* mutation or dysfunction-induced succinate accumulation results in multiple cancers and non-cancer diseases. The mechanistic studies show that succinate activates hypoxia response and other signal pathways via binding to 2-oxoglutarate-dependent oxygenases and succinate receptors. Recently, the increasing knowledge of ribonucleic acid (RNA) networks, including non-coding RNAs, RNA editors, and RNA modifiers has expanded our understanding of the interplay between SDH and RNA networks in cancer and other diseases. Here, we summarize recent discoveries in the RNA networks and their connections to SDH. Additionally, we discuss current therapeutics targeting SDH in both pre-clinical and clinical trials. Thus, we propose a new model of SDH–RNA network interaction and bring promising RNA therapeutics against SDH-relevant cancer and other diseases.

## 1. Introduction

Succinate dehydrogenase (SDH) is a mitochondrial enzyme present in supporting metabolic function through the tricarboxylic acid cycle (TCA cycle) and the electron transport chain (ETC). The enzyme works by catalyzing succinate to fumarate by oxidation in the TCA cycle, then ubiquinone is reduced to ubiquinol in the ETC [1]. As a part of the TCA cycle, SDH gains electrons and transfers them through the four subunits (SDHA, SDHB, SDHC, SDHD) and continues this electron transfer through the ETC as complex II. The electrons from FADH_2_ and reduced ubiquinone are transferred to complex III to continue the production of adenosine triphosphate. This produces the energy for the cell. With the regulation of this enzyme through its various complexes, the cells are able to perform cellular respiration, hypoxic response, and other cellular activities such as gene expression. However, altered SDH activity could give rise to disease and cancer development due to reduced electron flow, increased oxygen toxicity, and accumulated succinate. Due to the various subunits within the SDH complex, the difference in functionality can be responsible for these metabolic changes. In some human cancer cells, SDH demonstrates tumor-suppressive properties by inactivating hypoxia-inducible factor 1α (HIF-1α) via reduced succinate [2]. Additionally, the subunits of SDH can interact with ribonucleic acid (RNA) regulatory networks including non-coding RNAs, RNA-editing enzymes, RNA-modifying enzymes, transcription factors, and small molecules. A consequence of RNA modifications and deregulation of non-coding RNAs is the ability to act as tumor suppressors or oncogenes and alter gene expression, dysregulate cell signaling pathways, and alter cell metabolism [3,4]. Among them, non-coding RNAs can target SDH and contribute to complex dysfunction [5,6]. Additionally, SDH can be influenced by non-coding RNAs that are regulated by RNA-editing [7] and RNA-modifying enzymes [8] as well as transcription factors that have been found to contribute to various cancers. To combat the effects induced by *SDH* mutations or metabolic dysfunctions, multiple molecules including SDH inhibitors and activators are evaluated in current pre-clinical models and clinical trials. Here, we summarize the SDH-relevant physiological and pathological mechanisms as well as diseases including cancer. Additionally, we discuss innovative ways that RNA networks influence SDH state and promising strategies for targeting the SDH complex.

## 2. Succinate Dehydrogenase-Associated Genes and Protein Structures

### 2.1. SDH Complex-Associated Genes

**Figure 1:**
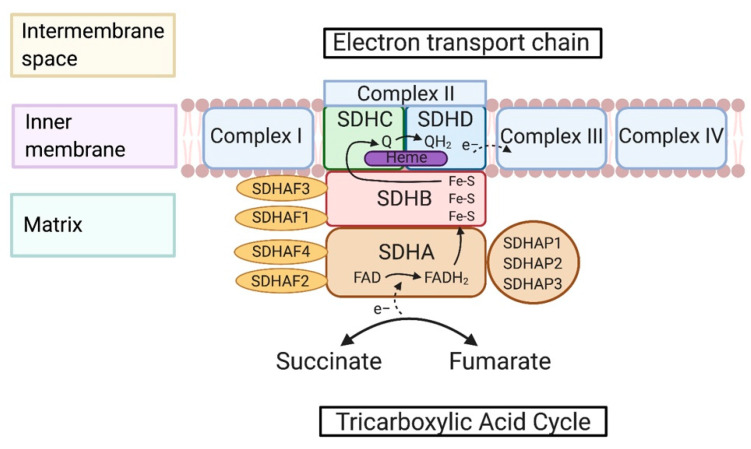
Structure, maturation, and assembly of succinate dehydrogenase (SDH) complex. The SDH complex, or mitochondrial complex II, sits within the inner mitochondrial membrane and is included in both the tricarboxylic acid cycle (TCA) and the electron transport chain (ETC). Succinate is an enzyme that is a part of the TCA cycle and is oxidized to fumarate through SDH; this is also present in the reverse reaction. From the oxidization, two electrons are transferred to subunit A to protonate FAD to FADH_2_ and release two electrons to the Fe-S clusters housed within subunit B. Assembly factors *SDHAF1* and *SDHAF2* assist in the maturation of subunits A and B. *SDHAF1* provides Fe-S clusters to SDHB and *SDHAF2* works in conjunction with dicarboxylate to stabilize the active site of SDHA. The next subunits, C and D, house heme and are responsible for ubiquinone reduction to ubiquinol. From here, ubiquinol is transferred to complex III of the ETC. The three known pseudogenes of SDHA, *SDHAP1-3*, are also included, whose metabolic function is still unknown.

The SDH complex is composed of four subunits that are encoded through nuclear genes: SDHA-D in mammals, SDH1-4 in yeast, and SDH1-8 in plants (Figure 1). Each subunit of the complex functions through assembly genes including *SDHAF1, SDHAF2, SDHAF3*, and *SDHAF4* or *SDH5, SDH6, SDH7,* and *SDH8* in yeast. *SDHAF2* is an important assembly factor of flavination of SDHA, needed for the SDH complex to be functional. *SDHAF2* works in conjunction with dicarboxylates of the TCA cycle by stabilizing the active site of SDHA [9]. SDHAF1 provides iron-sulfur (Fe-S) clusters for SDHB by first binding then recruiting the iron-sulfur cluster co-chaperone protein HscB (HSC20) [10]. *SDHAP1, SDHAP2,* and *SDHAP3* are pseudogenes that are a part of the SDHA complex (refer to GeneCards). Recently, it was found that lncRNA SDHAP1 upregulated the expression of EIF4G2 by reducing miR-4465 levels in ovarian cancer cells [11]. This suggests that the pseudogenes may regulate gene expressions through sponging microRNAs [12]. Further study of regulation by *SDHAP1-3* in the SDH complex could be beneficial for understanding the functions of the SDH complex that is beyond metabolic reactions.

### 2.2. Maturation and Assembly of the SDH Complex

The SDH complex is assembled through different complex genes (Figure 1). *SDHA* is responsible for the catalyzation of succinate to fumarate. In order for SDHA to be functional, it is dependent on sufficient flavin adenine dinucleotide (FAD) levels because it is a cofactor. Advantages of FAD cofactor binding to SDHA include increased redox potential to permit sufficient catalytic activity and stability of the overall complex [13]. It has been found that *SDHAF4* can serve as a chaperone of flavinylated *SDHA* by direct interaction prior to SDHA-SDHB complex formation by blocking excess reactive oxygen species (ROS) production [14]. The next subunit is *SDHB,* which stores Fe-S proteins; these proteins help transfer electrons from FAD to ubiquinone. Frataxin is a mitochondrial protein, and its deficiency can lead to Friedreich’s ataxia and iron accumulation in the mitochondria [15]. Frataxin deficiency can reduce the activity of Fe-S proteins which as a result can compromise SDH function [16]. *SDHAF1* and *SDHAF3* support SDHB maturation by transferring Fe-S clusters and bypassing respiratory distress and supporting respiratory growth by shielding SDHB from the oxidants [17]. The final step in the assembly of the complexes includes anchorage of SDHC and SDHD. This anchorage serves as the site for ubiquinone binding and ubiquinone reduction to ubiquinol. The heme b also sits in this domain but has been noted to not have a significant role in catalysis [18]. In mammalian structures, the heme b supports the structure of the membrane anchorage domain [19].

### 2.3. Metabolic Reactions of the SDH Complex

The SDH complex plays a vital role in cell metabolism considering its participation in the TCA cycle and ETC. This functional unit allows for the maintenance of ROS. SDH is responsible for oxidizing succinate to fumarate through the FAD redox reaction in the TCA cycle. There are multiple electron transfer pathways that allow the TCA cycle and ETC to function. Electrons are transferred to the three Fe-S clusters throughout the SDH complex (complex II), and these clusters start to transfer the electrons to ubiquinone. This is in preparation for ubiquinone reduction. Electrons are transferred from the Fe-S clusters to the ubiquinone pool which allows electron transfer between complex II and complex III [20]. Complex II is responsible for the reduction of ubiquinone to a semiquinone intermediate to ubiquinol. Heme also has many roles within the cell and serves as a prosthetic group for mitochondrial respiratory complexes. As discussed before, heme does not have a major role in catalysis, but it is essential for SDH complex assembly. In mammalian cells mutations in *SDHC H127A* and *SDHC H127Y* resulted in the decreased level of SDHC, a decrease in enzyme activity, and inhibition of complex II assembly formation [21]. The ETC acts as a proton gradient with proton pumping through complexes I, III, and IV. Although complex II is not involved in proton pumping, there is internal protonation involved with catalysis.

## 3. Pathogenesis of Succinate Dehydrogenase-Relevant Diseases and Mechanisms 

### 3.1. Cancers

**Figure 2:**
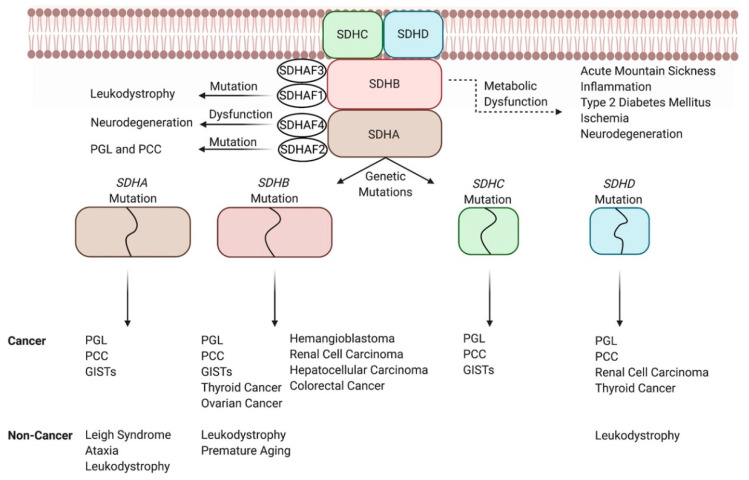
Genetic and metabolic dysfunction of the SDH complex and its link to diseases. For instance, *SDHB* mutations lead to the development of paraganglioma (PGL), pheochromocytoma (PCC), ovarian cancer, colorectal cancer, gastrointestinal tumors (GISTs), and other cancers. Similarly, *SDHB* mutations can cause non-cancer diseases such as leukodystrophy and premature aging. Another way that abnormal SDH activity causes the disease is through its metabolic dysfunction, as seen with decreased activities of SDHA and SDHB through epigenetic modification causing acute mountain sickness.

Early studies linked SDH complex dysfunction with cancer, evidenced specifically by studies that showed that *SDHB* [22], *SDHC* [23,24], and *SDHD* [22] mutations increased superoxide anion release (oxidative damage) which led to cells undergoing apoptosis or transformation. Recently, SDH is classified as a tumor suppressor, mostly due to two well-known abnormalities that it experiences, which allow for oncogenesis (Figure 2). First, SDH inactivation led to accumulation of succinate, which competitively inhibits HIF-α prolyl hydroxylase domain (PHD) and leads to the stabilization of HIF [25]. Stabilized HIF increases malignant cell proliferation by promoting angiogenesis [25] and ROS production [2]. Increased levels of succinate also led to the production of ROS by inhibiting α-ketoglutarate-dependent enzymes [26] and increasing the reverse flow of electrons from complex II to complex I [27]. Furthermore, the accumulation of succinate leads to increased histone methylation via binding directly and inhibiting histone demethylase JumonjiD3, which enhances epigenetic changes and oncogenic transformation [28]. We will discuss mutations in the *SDH* complex that reconfigure the aforementioned cellular mechanisms to induce tumorigenesis for certain cancers and their roles in other diseases.

#### 3.1.1. Paraganglioma and Pheochromocytomas

Mutations in *SDHB* [29], *SDHC* [30], and *SDHD* [31] have been implicated in causing two rare tumors in the autonomic nervous system known as paraganglioma (PGL) and pheochromocytoma (PCC) [32]. All of these specific genetic mutations are known to increase ROS production [22], which leads to DNA damage and tumorigenesis [33]. Interestingly, *SDHB* [22] and *SDHD* [34] mutations increased ROS production, which led to the onset of PGL and PCC through the stabilization of HIF mode of action. Mutations in all four subunits of *SDH* encoding genes have been shown to cause PCC and PGL through the inhibition of the histone demethylation route [28]. Interestingly, the same study found that *SDHB* mutations had a stronger effect in inhibiting histone methylases which allowed for increased hypermethylation [28]. This could explain why *SDHB*-mutated PGL and PCC are more malignant than when caused by mutations in other SDH-encoding subunits [35]. More recently, a group of researchers found that mutations of *SDHAF2* alone [36] were linked to PGL and PCC, despite research suggesting that SDHAF2 does not cause PGL and PCC through the inhibition of histone demethylation way of action [28]. However, no mechanism has yet been discovered. It is important to note that PGL and PCC are the tumor types most commonly associated with inheritance/germline mutations, specifically in the aforementioned SDH subunits [37], which highlights the dynamic role germline and somatic mutations within the SDH complex have in causing cancers and the importance of genetic counseling for the former.

#### 3.1.2. Other Cancers

PGL and PCC are most commonly associated with *SDH* mutations, but other cancers may also arise due to either SDH mutations or reduced *SDH* activities (Figure 2). For example, SDHB silencing led to increased levels of HIF-1α and adenosine monophosphate-activated protein kinase which promoted metastasis in ovarian cancer [38]. Likewise, the decrease in SDHB in hepatocellular carcinoma increased its malignancy through the Warburg effect and increased expression of epithelial–mesenchymal transition-related markers [39]. In colorectal cancer, wild-type *SDHB* has been shown to increase expressions of tumor suppressors such as phosphatase and tensin homolog (PTEN), caveolin-1, and cullin-5 to arrest the cell cycle, but its mutation led to increased cell division [40]. Carney–Stratakis syndrome, caused by germline mutations in *SDHA-B-C-D*, has been linked to causing PGL and GIST [41,42]. Specifically, SDH deficiency promoted DNA hyper-methylation at sites near the fibroblast growth factor 4 and tyrosine-protein kinase kit oncogenes, which led to their activation and the onset of GIST [43]. Although specific mechanisms such as the one elucidated for GISTs are not yet available, research has implicated *SDHB* and *SDHD* [44] mutations in renal cell carcinoma and thyroid tumors, SDHB defects [45] in pituitary adenomas, and loss of SDHB [46] in hemangioblastoma. The lack of established mechanisms for these cancers is highlighted specifically in the study attributing *SDHB* mutation to hemangioblastoma. Overall, there are few cases of hemangioblastoma, with only 19 of the total 35 patients testing positive for *SDHB* mutation [46]. The study stops short of conducting other experiments such as epigenetic analysis. Collectively, these issues do not allow for the relationship between SDHB inactivation and hemangioblastoma to be fully elucidated and similar issues arise when studying other *SDH* mutations and other cancers. Altogether, our understanding of the role of SDH between PGL and PCC and other cancers varies, but further research could provide therapeutic targets of SDH against cancer.

### 3.2. High-Altitude Illness (Acute Mountain Sickness)

Acute mountain sickness (AMS) is a result of the decreased partial pressure of oxygen at higher altitudes that causes tissue hypoxia [47]. The effects of AMS-induced hypoxia on mitochondrial function has been studied [48], with a consensus that oxidative damage increases with high altitude [49]. This is due to high altitude exposure increasing ROS production at complex I and complex III of the ETC, due to reduced electron flow [49,50]. Interestingly, Lu et al. compared AMS resistant individuals to AMS susceptible individuals and found that those resistant to AMS decreased plasma succinate levels through attenuation of SDHA and SDHB along with succinate-CoA ligase [51] (Figure 2). Succinate-CoA ligase is a key enzyme involved in converting succinyl-CoA to succinate through the following subunits: succinate-CoA ligase GDP/ADP-forming subunit alpha (SUCLG1), succinate-CoA ligase GDP-forming subunit beta (SUCLG2), and succinate-CoA ligase ADP-forming subunit beta (SUCLA2) [52,53]. The study suggested that the aforementioned genes were silenced due to exposure to high altitude, with the exact mechanisms yet to be discovered [51]. Similarly, PGL exhibits the same environmental and genetic crosstalk seen in AMS as prolonged exposure to high altitudes led to a mutation in *SDHB* which caused PGL in patients [54]. A clear link is shown between high-altitude, SDH, AMS, and cancer, and further research could be beneficial for a better understanding of this intricate connection.

### 3.3. Inflammation

SDH has shown the capability to play key roles in pro- and anti-inflammatory signaling (Figure 2). For instance, lipopolysaccharide-stimulated interleukin 1 beta (IL-1β) release in macrophages promotes inflammation via increased succinate levels, resulting in PHD-mediated HIF-1α accumulation [55] or SDH-catalyzed ROS production [56]. IL-1β can also be produced independent of HIF-1α through the inflammatory release of succinate by macrophages which activates succinate receptor 1 (SUCNR1)/G-protein coupled receptor 91 (GPR91) and enhances the adverse effects of rheumatoid arthritis [57]. Conversely, inflammatory mononuclear phagocytes increase succinate levels to stimulate SUCNR1/GPR91 which allows uptake of succinate by neural stem cells, and their subsequent anti-inflammatory phenotype through reduction of IL-1β [58]. As exhibited, succinate has a role in the inflammatory response of cells, and further research can provide therapeutic options as seen with SUCNR1/GPR91 inhibitors for rheumatoid arthritis [57].

### 3.4. Neurodegenerative Disease

SDH has an important role in electron flow [19,59], exemplified by SDH activity causing electron carriers such as nicotinamide adenine dinucleotide (NAD) to not be oxidized which in turn leads to a decrease in electron flow to complex III and ubiquinone, and the production of ROS [60]. Mutations in *SDHA* have been shown to lead to this mechanism and the development of Leigh syndrome [61,62]. Other mutations of *SDH* subunits that have been implicated in neurodegenerative diseases include *SDHA* causing ataxia [63] and *SDHA* [64], *SDHB* [64], *SDHD* [65], and *SDHAF1* [66] leading to leukodystrophy, yet these studies have been limited to few patients (Figure 2). Nevertheless, as stated in a recent review paper [67], succinate is linked with the mammalian target of rapamycin (mTOR) [68], a kinase involved with a plethora of neurodegenerative diseases [69]. This provides interest for further studying roles of succinate in neurodegenerative diseases, especially since SDHAF4 has been shown to stabilize succinate accumulation, increase mitochondrial SDH activity, while limiting ROS production and preventing neurodegeneration in drosophila [14].

### 3.5. Diabetes

The succinate mechanism of insulin release states that high mitochondrial levels of succinate produce mevalonic acid, which triggers insulin release in pancreatic islet cells (refer to source paper for full mechanism) [70]. Proteomic analyses of type 2 diabetes mellitus (T2D) patients with chronic hyperglycemia showed that increased glucose levels led to inhibition of the ETC. This is due to decreased expression of SDH along with citrate synthase and fumarate hydratase in the mitochondria, which led to decreased insulin release of pancreatic β-cells [71]. While succinate levels decrease in the mitochondria, circulating levels of succinate have been shown to increase in T2D patients [72]. This circulating succinate can accumulate in the diabetic kidney, leading to succinate induced activation of GPR91, a receptor that activates the renin-angiotensin system [73]. The activation of renin-angiotensin system in diabetic rats has been linked to induced hypertension and nephropathy [74]. Further research of succinate’s role in diabetes is encouraged as a link has already been established.

### 3.6. Ischemia-Reperfusion Injury

Succinate is known to accumulate during cardiac ischemia, which is then consumed during reperfusion and leads to oxidative damage due to increased ROS production [75]. A leading model to explain this phenomenon is the upregulation of glycolysis and the TCA cycle, known to occur during ischemia. These two processes drive the conversion of glutamate to 2-oxoglutarate, which is then synthesized into succinate through succinate-CoA ligase [76]. Barth syndrome, a genetic disease known to cause cardiomyopathy, leads to the loss of SDH in cardiac tissue and a subsequent increase in ROS production [77]. Although cardiac ischemia and Barth syndrome are not caused by succinate, their effect on succinate has unfavorable consequences, and research of the effect of non-SDH induced diseases on SDH dysfunction is a topic warranting further analysis. 

## 4. A Network of RNA Regulators Interacting with Succinate Dehydrogenase

### 4.1. Non-Coding RNAs

**Figure 3:**
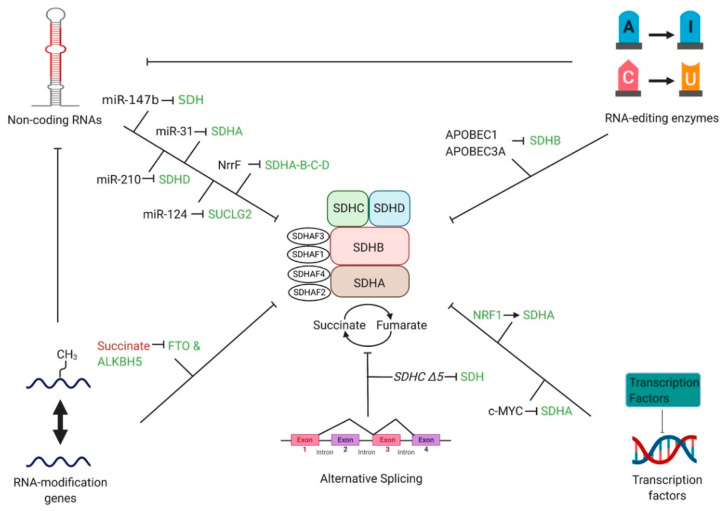
A network of RNA regulators interacting with succinate dehydrogenase. Different proteins/processes affecting the transcription/translation of the SDH complex are shown. For example, the RNA-editing enzyme APOBEC3A decreases the expression of SDHB. Similarly, the non-coding RNAs miR-31 and miR-210 decrease SDHA and SDHD activity, respectively. It is important to note that the interplay between RNA-editing enzymes and RNA-modification genes with non-coding RNAs has been established, but the possible role of these regulators and their effect on SDH has yet to be discovered and warrants intrigue. Regulations are signified with inhibition (┤) and promotion (→) together with upregulation (red lettering) and downregulation (green lettering).

Non-coding RNAs are transcribed RNAs known for their regulatory function on mRNA [78] and their ability to affect several cellular processes to influence disease states [79,80,81,82]. Non-coding RNAs include microRNAs (miRNAs), small non-coding RNAs (sRNAs), long non-coding RNAs (lncRNAs), and circular RNAs among others [78]. Numerous non-coding RNAs, especially miRNAs, have been shown to target SDH and lead to disease. In lung cancer, increased expression of miR-147b [6] downregulates SDH and leads to drug tolerance to epidermal growth factor receptor tyrosine kinase inhibitor, while upregulated miR-210 [5] lowers the enzymatic activity of SDHD to stabilize HIF-1α. miR-31 was found to suppress SDHA expression in induced pluripotent stem cells to initiate the Warburg effect [83]. An expression profile study found that miR-124 can inhibit the conversion of succinate to succinyl-CoA by downregulating SUCLG2 [84], showing that other miRNAs could affect SDH and perhaps other diseases. The sRNA, NrrF, after binding with the Hfq protein, binds to the sdhCDAB mRNA transcript in *N. meningitidis* and upregulates SDHA, SDHB, SDHC, and SDHD [85]. Crosstalk between miR-488-3p and the lncRNA Cerox1 regulates mitochondrial complex I activity, showing promise that interactions between non-coding RNAs may affect the SDH complex [86]. Altogether, non-coding RNAs have been shown to affect SDH levels and even modulate disease progression (Figure 3).

### 4.2. RNA-Editing Enzymes

RNA editing is a posttranscriptional mechanism that involves the alteration of RNA sequences through insertions, deletions, or substitutions done by enzymes [87]. To date, the two well-known RNA editing enzymes, adenosine deaminases acting on RNA (ADARs) [88,89] and apolipoprotein B mRNA editing enzyme, catalytic polypeptide-like (APOBEC) [90], cause adenine to inosine (A-to-I) and cytidine to uracil (C-to-U) editing on RNA transcripts, respectively. Hypoxic conditions in peripheral blood mononuclear cells, along with their differentiation into macrophages, led to increased C-to-U editing by apolipoprotein B mRNA editing enzyme catalytic subunit 1 (APOBEC1), a member of the APOBEC family. This caused a nonsense mutation and the inactivation of the *SDHB* gene [91] (Figure 3). Continuously, Baysal’s lab demonstrated for the first time that another APOBEC family member APOBEC3A modifies SDHB RNA in monocytes and macrophages via a C-to-U editing mechanism that is activated by hypoxia or interferons [92]. Section 3 of this review describes different ailments such as cancer caused by mutations in *SDH* subunits, including *SDHB*, which signifies the importance of studying the effects of RNA-editing enzymes on SDH. It is important to note that RNA editors can also affect the processing of non-coding RNAs, as seen with increased A-to-I editing in miR-376 which changes the targets of miR-376 [93]. The role of RNA editors in miRNAs and other non-coding RNAs that subsequently affect SDH is a topic that is yet to be investigated but is an exciting prospect. This is of significant interest to further study the effects of APOBEC and ADAR due to its wide-ranging effects on SDH-affecting genes.

### 4.3. RNA-Modification Genes

N^6^-methyladenosine (m^6^A) modification of mRNA is the most abundant RNA modification and plays a pivotal role in determining gene expression [94]. Fat mass and obesity-associated protein (FTO) and alpha-ketoglutarate-dependent dioxygenase AlkB homolog 5 (ALKBH5) are two well-characterized m^6^A erasers that reverse the actions of writers for m^6^A through demethylation [95]. These m^6^A erasers are also identified as 2-oxoglutarate-dependent oxygenases. Inactivating *SDH* mutations induces succinate accumulation, a product that competes with 2-oxoglutarate and hence may inhibit the expression of FTO and ALKBH5 [96]. Similar to RNA editors, the role between RNA-modifiers and non-coding RNAs has been elucidated (and its role in causing cancer) [97] (Figure 3). It is crucial to investigate whether an interplay exists between RNA modifiers and non-coding RNAs that impact SDH activity. Overall, it is critical to study the relationship between SDH complex dysfunction and m^6^A erasers as the latter’s depletion has been linked to diseases such as male-infertility [98]. 

### 4.4. Transcription Factors

Transcription factors play an intricate role in gene expression as they activate or repress transcription of DNA into RNA [99], and its misregulation has been implicated to cause disease [100]. Nuclear respiratory factor 1 (NRF1) silencing attenuated SDHA expression at transcriptional levels in cardiac cells, causing complex II dysfunction, which had a final effect of decreased PHD activity leading to hypoxia response through HIF-1α stabilization [101] (Figure 3). MYC enhances S-phase kinase associated protein 2 (SKP2) activity, a proteasome that degrades SIRT3 deacetylase, which led to increased acetylation of SDHA and its subsequent silencing [102]. MYC-mediated dysfunction of SDH activity led to succinate accumulation and increased H3K3me3 modification on histones [102]. Overall, this modification promoted tumor progression in Burkitt’s lymphoma cell lines, but the specific genes that were downregulated to cause this effect were not mentioned [102]. Here, we show that increased and decreased activity of MYC and NRF1, respectively, modulate disease states through transcriptional effects on SDH subunits. 

### 4.5. Alternative Splicing

The manipulation of the order of exons in mature mRNA due to alternative splicing mechanisms is well known to alter gene expression [103]. In SDH, the *SDHC* ∆*5* isoform which lacks exon 5, formed by alternative splicing mechanisms, decreased SDH activity by 40%. This led to a notable increase in the production of ROS [104] (Figure 3). However, another isoform of *SDHC* (∆*3*) had a marginal effect on SDH activity and only minimal ROS production [104]. Nevertheless, alternative splicing in SDH subunits provides another plausible explanation of how SDH subunit dysfunction can lead to disease. 

## 5. Treatment against Succinate Dehydrogenase Dysfunction

### 5.1. Small Molecules Inducing or Blocking SDH Activity

While the study of mitochondrial regulation and SDH has been around for over 100 years, still little is known about the details of the molecular pathways taken by SDH activators and inhibitors. SDH is regulated through the genetic level and three distinct binding sites. These sites have been exploited for use from fungicides to tumor cell regulation.

#### 5.1.1. SDH Inhibitors

**Figure 4:**
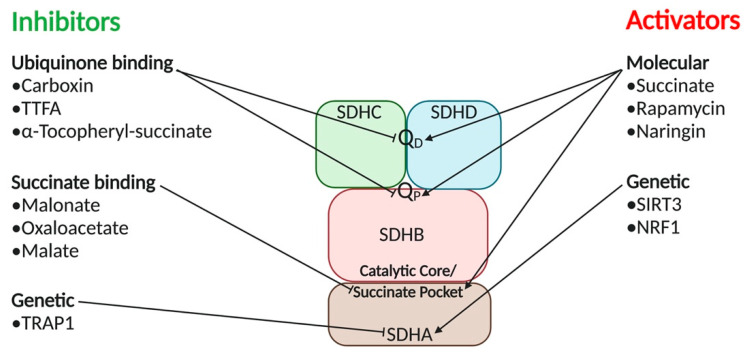
Strategies for Targeting Succinate Dehydrogenase Complex. Ubiquinone binding inhibitors carboxin, TTFA, and α-Tocopheryl succinate bind and deactivate SDH at the proximal Q_P_ and distal Q_D_ ubiquinone binding sites. Ubiquinone binding inhibitors disrupt the reduction of ubiquinone to ubiquinol, a key step in the ETC. Succinate binding inhibitors malonate, oxaloacetate, and malate bind at the catalytic core or succinate pocket of SDH. Succinate pocket inhibitors are typically intermediates from the TCA cycle that modulate SDH activity based on cellular needs. Genetic inhibitor TRAP1 is a mitochondrial chaperone that inhibits SDH functions causing hypoxic environments that stimulate tumorigenesis. SDH activators such as succinate, Rapamycin, and Naringin function as small molecules activators of SDH. Additionally, genetic activator *SIRT3* regulates the deacetylation of SDHA. NRF1 is an oxygen-sensing protein that binds to SDH genetic promoters when there is a lack of oxygen to limit ETC energy production until suitable oxygen conditions are resumed.

There are two distinct classes of metabolic SDH inhibitors based on where the inhibitor binds. SDH inhibitors either bind in the ubiquinone pocket at the proximal (Q_p_) or distal (Q_D_) binding sites, or in the succinate pocket [105]. Briefly, binding to the ubiquinone pocket affects the reduction of ubiquinone to ubiquinol which is a part of the ETC. Additionally, binding to the succinate pocket influences the enzymatic activity of SDH inside of the TCA cycle [106] (Figure 4).

Carboxin is a ubiquinone inhibitor commonly used as a fungicide. Thenoyltrifluoroacetone (TTFA) is another ubiquinone SDH inhibitor that was originally thought to be a chelating agent [107]. After the development of 19 SDH inhibitors, it was determined that all of them share a carbonyl center, a conserved amide function, and an amine functional group. Yao et al. took these common groups and applied in silico library design and pharmacophore mapping to create potential novel inhibitors. A16c showed to be the most promising inhibitor from this study, with increased potency against standard fungal targets [108]. Genetic regulation of SDH subunits has also been documented. SDHB expression has been shown to be lowered in maize leaves related to the amount of light present [109]. This is due to the reduced need for energy from the TCA cycle when photosynthesis is occurring. *SDH2-3* gene function was downregulated in response to the lower expression of the phytochrome interacting factor 3 gene, which is involved in several developmental processes of plants. SDH inhibitors have been used as fungicides since the 1960s, but recently there has been a surge in SDH inhibitors as a cancer therapeutic strategy.

The human application of SDH inhibitors is still in its early stages. SDH inhibitors functioning as fungicides have been difficult to bring to human use mainly due to the cellular similarities between fungi and humans causing cytotoxicity. TTFA, the original SDH inhibitor, was shown to induce cell apoptosis in neuroblastoma cells [110]. Alpha-Tocopheryl succinate, a similar compound to vitamin E, is another SDH inhibitor shown to induce cell apoptosis in cancer cell lines via binding to the Q_P_ and Q_D_ [111]. Malonate, a synthetic compound, has been used as an SDH inhibitor to limit reperfusion injury and infarct size in mice and pig hearts [112]. TCA cycle intermediates oxaloacetate and malate are strong inhibitors of SDH binding to its catalytic site [113]. Mitochondrial chaperone tumor necrosis factor receptor associated protein 1 (TRAP1) has been identified as an inhibitory SDHA binding protein that is overexpressed in many types of cancers [114]. TRAP1 binding to SDHA decreases SDH activity and limits mitochondrial-dependent respiration in cancer cells. The reduced SDH activity-induced succinate accumulation leads to hypoxic response via activating HIF-1α. This scenario allows tumor cells to survive nutrient-depleted environments and oxidative stress [115].

#### 5.1.2. SDH Activators 

SDH can be activated on both metabolic and genetic fronts. On the metabolic front, succinate activates the catalytic function of SDH through dissociating any SDH inhibitors such as oxaloacetate and malate in the TCA cycle [116]. Rapamycin is currently being studied as a potential treatment against SDH deficiency. Exposing flies with mutated *SDHA* and *SDHB* to rapamycin improved the enzymatic activity of SDH through the inhibition of the mTOR pathway [117]. In addition, naringin, a bioflavonoid found in citrus fruit peel, restored SDH enzymatic activity that was impaired by D-galactose via influencing ROS [118] (Figure 4). In the ETC, there is a balance of phosphorylation and acetylation contributing to the activity of SDH [119]. The less acetylation and phosphorylation present, the more active SDH is. Sirtuin-3 (SIRT3) is the primary deacetylase for SDHA and is the main contributor to the catalytic ability of SDH inside the ETC [120]. This NAD-dependent enzyme mediates the reversible acetylation of SDHA through post-translational modifications (Figure 4). NRF1 has been shown to bind to the promoters of the *SDHA* and *SDHD* genes in aerobic rat cardiac cells resulting in increased expressions of SDHA and other subunits of SDH complex. This binding is a response to a lack of oxygen where the cell relies on glycolytic ATP production until suitable conditions resume for ETC production of ATP [101]. Additionally, reduced SDH expression is inversely related to promoter methylation of *Sdh1-2* which encode for SDHA in maize under anoxic conditions [121].

### 5.2. CRISPR

Clustered regularly interspaced short palindromic repeats (CRISPR) is a powerful gene-editing tool [122]. Recent studies have shown the effects of editing different SDH subunits and the effect on certain diseases. For example, the downregulation of SDHC using CRISPR in breast cancer cell lines correlated with a smaller amount of the cancer cells undergoing epithelial to mesenchymal transition [123].

## 6. Pre-Clinical Models and Clinical Trials for Succinate Dehydrogenase

### 6.1. Approaches to Measuring SDH Activity

SDH activity can be measured using many different approaches. The Succinate Dehydrogenase Assay Kit is a colorimetric assay designed to measure SDH activity in tissue culture or purified mitochondrial samples. The assay detects the production of fumarate from succinate by SDH. Fumarate hydratase converts fumarate to malate and then malic dehydrogenase converts malate to pyruvate and nicotinamide adenine dinucleotide phosphate (NADP^+^) to nicotinamide adenine dinucleotide phosphate hydrogen (NADPH) which is detected spectrophotometrically [124]. SDH activity can also be measured using a redox dye such as an artificial electron acceptor reporter module 2,6-dichlorophenolindophenol (DCPIP). In a conversion from succinate to fumarate, SDH transfers the electron to oxidized DCPIP, which changes the color from blue to the pink or colorless product. By adding surface-enhanced Raman scattering, results can be specified and a more accurate level of SDH activity can be determined [125]. SDH levels have also been measured using a microphotometric assay coupled with human muscle samples [126]. This assay uses nitro blue tetrazolium (NBT) to bind to mitochondria and function as a final electron acceptor. The enzymatic activity of SDH can then be measured by taking absorbances at certain time points to create a reaction curve [127].

### 6.2. Pre-Clinical Models

SDH has been studied in plants since the 1960s. Plants are advantageous in that they have all four SDH subunits and SDH serves a similar role in the TCA cycle and ETC as in humans. The disadvantage of plants is that they contain four accessory subunits, SDH5–SDH8 [128]. These accessory subunits are not conserved in humans and their specific functions are unknown. The plant SDH3 and SDH4 lack sequences that help it bind to SDH1. Similar sequences have been found in plant SDH6 and SDH7 that could help stabilize the complex. SDH5 is a more hydrophobic subunit that interacts with SDH2 and SDH4, but its function is unknown. SDH8 is the least understood accessory subunit. The subunit is only 4.9 kDa in size and does not show any similar sequences in the current genome mapping to any other SDH subunits [129].

Yeast contains the same subunits as other eukaryotic organisms. Yeast is advantageous over other organisms because both respiratory and fermentative metabolism can be observed in the same model. Yeast has also been shown to recreate human *SDH* mutations specifically in *SDHB* and *SDHC*. This ability allows researchers to study the specific mutations in detail in a preclinical model not typically available in other organisms [130].

Most studies conducted with *Escherichia coli* focus on the adaptation to changing oxygen environments and the gene expressions related to the changes. Through the *E. coli* study, it has been determined that the alpha-ketoglutarate dehydrogenase and succinyl coenzyme A synthase are regulated by SDHC. These enzymes are important in the TCA cycle component of SDH metabolism. *E. coli* as a preclinical model is beneficial because of its easily manipulated genome [131]. 

*Caenorhabditis elegans*, commonly referred to as the nematode, is beneficial as a preclinical model because of its thoroughly understood development, small genome, and short, complete life [132]. In SDH research, the nematode has been used to study mutations in *SDHB* leading to tumorigenesis. The study found that the ubiquinone-binding site of SDH became a significant source of superoxide, but the effects were abrogated with the administration of antioxidants [133].

The transgenic mouse is one of the most advanced preclinical models to investigate tumorigenesis and other pathologies contributed to *SDH* mutations. Piruat and Millan-Ucles demonstrated knockout mouse models for each SDH subunit gene and how it affected development [134]. Other mouse models have also linked *SDH* mutations to tumorigenesis through oxygen-depleted environments and epithelial to mesenchymal transition [135]. Preclinical models can show a general direction on how a clinical trial will go and account for metabolic environments not replicable in vitro. These models are a critical testing ground for higher stakes clinical trials.

### 6.3. Clinical Trials

Clinical trials associated with SDH dysfunction range from advanced cancers to neurodegeneration (Table 1). The broader study of mitochondrial dysfunction also accounts for many clinical trials where SDH dysfunction plays a role.

Temozolomide was tested to observe the effects on *SDH*-mutant/deficient GISTs (NCT03556384). Temozolomide is an alkylating agent that has already been approved for use against glioblastoma multiforme and refractory anaplastic astrocytoma tumors. *SDH*-mutant/deficient GISTs show hypermethylation which leads to loss of proteins, specifically O^6^-methylguanine-DNA methyltransferase. The inhibition of this protein due to promotor methylation has shown to lead to effective use of alkylating agents in other cancer types [136]. The rationale was to use a known chemotherapy drug, temozolomide, against a type of tumor it had not been tested against. DNA methyltransferase inhibitor, Guadecitabine (SGI-110), was tested on wild type GISTs, pheochromocytoma, and paraganglioma associated with SDH deficiency in hereditary leiomyomatosis and renal cell carcinoma (NCT03165721). The rationale behind this studying is the methylation of SDH contributing to increased ROS and anaerobic environments causing cells to develop mutations. By inhibiting methylation, tumor growth and mutation can be slowed. Glutaminase inhibitor CB-839 was tested on SDH-deficient GISTs, SDH-deficient non-GIST tumors, triple-negative breast cancer, and others for their interactions with CB-839 and standard chemotherapy (NCT02071862). Glutamine metabolism has been shown to be upregulated in *SDHB* mutated cancers. Glutaminase-1 generates glutamate from glutamine which is then metabolized to α-ketoglutarate by glutamate dehydrogenase [137]. The α-ketoglutarate can then be used by the TCA cycle. This leads to an accumulation of succinate that stabilizes and activates HIF1 and HIF2 [138]. This reliance on glutamine metabolism by *SDH* mutant cancers has created an increased sensitivity to glutaminase inhibitors. The rationale behind the trial is to introduce the glutaminase inhibitor to regular chemotherapy as a new way to slow tumor growth.

The synergy between nivolumab and cabozantinib is being tested in SDH and fumarate hydratase deficient renal cell carcinomas (NCT03635892). Nivolumab is a monoclonal antibody that inhibits programmed cell death-1 (PD-1). In SDH deficient tumors, PD-1 receptor-ligand signaling is dysregulated due to hypoxic conditions [139]. This combination therapy with the tyrosine kinase inhibitor, cabozantinib, will target both programmed cell death signaling and vascular endothelial growth factors [140].

*SDH* mutation can lead to several different cancers including paragangliomas. However, there is little information on environmental and professional factors playing a role in cancer risk. This study is a cross-section of patients with the same sex, age, and gene affected without tumors. Environmental and professional conditions will be accessed and compared through an interview (NCT04481152).

Cancer-related SDH clinical trials look to target *SDH* mutation that leads to tumor progression. Many current therapies are ineffective against dysfunctional or mutated *SDH* subtypes of their parent tumor class. By continuing these trials, cancer is being targeted through a mechanism that affects both tumor progression and maintenance. By inhibiting the mutant *SDH* or restoring normal function, not only can tumor progression halt, but tumor recession can also take place.

Patient resistance to high altitude via their chemoreflex, a reflex associated with hyperventilation, was tested to find a link between altitude sickness and SDH functionality (NCT00202683). The rationale is that participants with an altered or pathogenic chemoreflex will be intolerant to high altitude and exhibit cerebral edema or pulmonary edema. While no results were published, the implications are that dysfunctional SDH subunits contribute to an inability of the carotid body to respond to changes in altitude. This now established relationship could inspire treatments with antioxidants or gene therapies.

The North American Mitochondrial Disease Consortium Patient Registry and Biorespiratory group aims to identify individuals with mitochondrial disorders and connect them with other current clinical trials for which they may be eligible. Additionally, patient tissue samples will also be cataloged and stored in a shared facility where several groups may have access to the samples to further their research (NCT01694940).

Macrophage mediated pro-inflammatory response is a common issue in diabetic and other related cardiovascular complications. Recent studies suggest that glutamine catabolism is involved in the activation of these macrophages through TCA cycle intermediates. The study will look at glutamine metabolism and levels of α-ketoglutarate, fumarate, and succinate (NCT04353869).

Non-cancer related SDH trials cover a wide variety of diseases. SDH trials related to mitochondrial disease cover many different subsets related to genetic conditions. The chemoreflex also shows SDH in the capacity of internal respiration. This wide range of trials shows the dependency of the human body on SDH.

## 7. Future Directions

In this study, we systematically summarized how the SDH complex interacts with the RNA networks to regulate the development of cancer and other diseases. There are several promising points that need to be investigated further in the future to better understand the mechanisms of SDH in pathogenesis.

First, it is of interest to study how SDH pseudogenes such as SDHAP1/2/3 interact with SDHA/B/C/D subunits and other molecules. Utilizing the cutting-edge cryo-electron spectroscopy [141] will facilitate our understanding of this interaction and further insight on cancer and disease development. Unexpectedly, SDHAP1 is classified as a lncRNA in ovarian cancer [11]. It indicates that SDHAP1/2/3 might play a role independent of SDH-enzyme based metabolic reactions.

The studies of an interplay between SDH complex and non-coding RNAs are still at an early stage. Among them, only a limited number of miRNAs have been studied. A large-scale screening of non-coding RNAs using (small) RNA-sequencing should be applied to this study. Additionally, studies for RNA editors of SDH such as C-to-U editing by APOBEC1 and APOBEC3A have received increasing notices recently. In addition to this direct APOBEC1/3A−SDH interaction, RNA editors can also manipulate non-coding RNA levels in cancers and non-cancer diseases [142]. Thus, how RNA editors regulate SDH indirectly via editing non-coding RNAs needs to be investigated in the future. In turn, SDH inactivation-induced succinate accumulation can induce the upregulation of miRNAs such as miR-210. It is of interest to study whether succinate accumulation can also influence the activity of RNA editors. We need to highlight that the interaction of the SDH−RNA network is dynamic due to the fact that some RNAs can play roles as messengers because they may be transmitted from one type of cell to another type of cell. For example, cell-free circulating exosomal miRNAs can transmit signals when they are delivered from the original site to the target site [143]. Similar to circulating miRNAs, succinate has been discovered to shuttle from hypoxic retina to oxygen-enriched tissue to transfer electrons [144]. Thus, this new module provides a possibility that the SDH−RNA interaction can be applied in different microenvironments when RNA and succinate are transmitted remotely.

Severe fungal infections to humans have caused many deaths in patients [145]. Most of the fungicides are designed to combat human fungal pathogens through inhibiting cellular mitochondrial respiration such as SDH activities in fungi [146]. SDH inhibitors targeting fungus infection has been difficult to incorporate into human application due to the similar SDH structures in human and fungal cells. Thus, the following two concerns need to be addressed when applying the SDH inhibitors in antifungal (antipathogen) treatment in humans. The applied SDH inhibitors can be transmitted to human cells leading to increased risk of tumorigenesis due to inactivating tumor suppressor SDH in human cells. Additionally, some pathogens such as Gram-negative bacterial product lipopolysaccharide-induced succinate accumulation activate HIF-1α and enhance the release of interleukin-1β in human macrophages [55]. Additionally, pathogen-derived succinate might also influence tumor initiation and progression in humans [116,147].

RNA-based therapeutics have received increasing attention recently due to its advantage of accuracy and specificity compared to conventional small molecules [148]. However, the delivery efficiency and off-target effect of RNA therapeutics need to be addressed properly before entering clinical trials. It is critical to improve the RNA delivery to targeted cells by using cutting-edge nanoparticles and ligand-conjugated carriers [149]. Simultaneously, exploring the distribution and functions of RNA-based-therapeutics in non-targeted cells (i.e., non-tumor cells in cancer) can facilitate the applications of RNA therapeutics entering clinical trials [150]. In the future, it is of importance to test the possibility of utilizing RNA-based therapeutics such as targeting non-coding RNAs, RNA editors, and RNA modifiers to conquer *SDH* mutation- or dysfunction-induced cancer and diseases.

## Figures and Tables

**Table 1:** SDH relevant clinical trials.

Study Title	Description	Dates (Start–Completion) and Status	Identifier and Study Type (Enrollment)
Cancers			
An Open-Label, Phase 2 Efficacy Study of Temozolomide (TMZ) In Advanced Succinate Dehydrogenase (SDH)-Mutant/Deficient Gastrointestinal Stromal Tumor (GIST)	Therapies already exist for advanced GIST, but they are not effective against *SDH* mutant subtypes of GIST. TMZ is already approved for the treatment of other glioblastoma tumors, but its effect on SDH mutant GIST has not been previously studied.	September 2018-September 2024 (Recruiting)	NCT03556384 Interventional (N.A.)
A Phase II Trial of the DNA Methyl Transferase Inhibitor, Guadecitabine (SGI-110), in Children and Adults with Wild Type GIST, Pheochromocytoma and Paraganglioma Associated with Succinate Dehydrogenase Deficiency and HLRCC-associated Kidney Cancer	To determine the overall response to SGI-110 in tumor growth and effects on the body. GIST is resistant to conventional radiation or chemotherapy treatments. Imatinib is the current standard of care but tumor-developed resistance and mutations are becoming more prevalent. SGI-110 is targeting these tumors by preventing DNA methylation and has shown to be effective against imatinib resistant GIST.	May 2017- February 2020 (Completed)	NCT03165721 Interventional (9)
Ph1 Study of the Safety, PK, and PDn of Escalating Oral Doses of the Glutaminase Inhibitor CB-839, as a Single Agent and in Combination with Standard Chemotherapy in Patients with Advanced and/or Treatment-Refractory Solid Tumors	Tumor cells have been shown to be dependent on glutamine for cellular respiration. Because this is a unique trait to tumors, this dependence serves as a potential therapeutic target. CB-839 is a highly specific inhibitor targeting glutaminase, the first enzyme involved in glutamine utilization. This study looks at the potency of this inhibitor across a wide range of tumors.	February 2015- March 2019 (Completed)	NCT02071862 Interventional (210)
A Phase 2 Open-Label Study of Nivolumab Combined with Cabozantinib in Subjects with Advanced or Metastatic Non-Clear Cell Renal Cell Carcinoma (CA209-9KU)	Nivolumab is a programmed cell death protein 1 inhibitor, and cabozantinib is a tyrosine kinase inhibitor. Both drugs are approved treatments against several types of metastatic kidney cancers, but there are limited data on combination treatments using these two drugs.	August 2018-August 2021 (Recruiting)	NCT03635892 Interventional (N.A.)
Impact of Environmental Exposures on Tumor Risk in Subjects at Risk of Hereditary SDHx Paraganglioma (PGL-EXPO-1)	By studying environmental and professional factors of patients with an *SDHx* mutation and comparing it to a patient with the same sex, age, and type of gene affected but no tumor progression, researchers hope to identify novel contributors to *SDHx* genetic mediated tumor progression.	January 2021-December 2022 (Not yet recruiting)	NCT04481152 Observational (N.A.)
Non-cancer			
Relationship Between Succinate Dehydrogenase Mutations and High-Altitude Illness Associated with Chemoreflex Failure	SDH dysfunction is known to cause hypoxia. At high altitudes where oxygen is limited, a cell already coping with SDH dysfunction would be overwhelmed. The chemoreflex causes hyperventilation when the pressure of oxygen falls in the blood. A dysfunctional chemoreflex can lead to pulmonary and cerebral edema at high altitudes.	March 2005-December 2006 (Completed)	NCT00202683 Observational (83)
North American Mitochondrial Disease Consortium Patient Registry and Biorepository (NAMDC)	The NAMDC is building an international network of researchers, patients, and data to help both the researcher and patient connect with the proper clinical trials and potential treatments.	December 2010-December 2025 (Recruiting)	NCT01694940 Observational (N.A.)
Targeting Glutamine Metabolism to Prevent Diabetic Cardiovascular Complications (GLUTADIAB)	This study has broad metabolic implications and serves as the starting point for several secondary studies. After glutamine metabolism is better understood in its role in the inflammatory response, several other factors will be analyzed including SDH-controlled intermediates. RNA modification will also be utilized to target monocytes.	June 2020- June 2022 (Not yet recruiting)	NCT04353869 Observational (N.A.)

## Abbreviations

ADARs	Adenosine deaminases acting on RNA
ALKBH5	Alpha-ketoglutarate-dependent dioxygenase AlkB homolog 5
APOBEC	Apolipoprotein B mRNA editing enzyme, catalytic polypeptide-like
APOBEC1	Apolipoprotein B mRNA editing enzyme catalytic subunit 1
APOBEC3A	Apolipoprotein B mRNA editing enzyme catalytic subunit 3A
A-to-I	Adenine to inosine
ATP	Adenosine triphosphate
CRISPR	Clustered regularly interspaced short palindromic repeats
C-to-U	Cytidine to uracil
DCPIP	2,6-dichlorophenolindophenol
DNA	Deoxyribonucleic acid
ETC	Electron transport chain
FAD	Flavin adenine dinucleotide
Fe-S	Iron-Sulfur
FTO	Fat mass and obesity-associated protein
GBM	Glioblastoma multiforme
GISTs	Gastrointestinal tumors
GPR91	G-protein coupled receptor 91
HIF-1α	Hypoxia-inducible factor 1 alpha
HRLCC	Hereditary leiomyomatosis and renal cell carcinoma
IL-1β	Interleukin 1 beta
lncRNA	Long non-coding ribonucleic acid
mTOR	Mammalian target of rapamycin
miRNA	Micro-ribonucleic acid
m^6^A	N^6^-methyladenosine
NAD	Nicotinamide adenine dinucleotide
NADP+	Nicotinamide adenine dinucleotide phosphate
NADPH	Nicotinamide adenine dinucleotide hydrogen
NBT	Nitro blue tetrazolium
NRF1	Nuclear respiratory factor 1
PCC	Pheochromocytoma
PGL	Paraganglioma
PD-1	Programmed cell death-1
PHD	HIF-α prolyl hydroxylase domain
PTEN	Protein and tensin homolog
Q_D_	Distal binding site in ubiquinone
Qp	Proximal binding site in ubiquinone
RNA	Ribonucleic acid
ROS	Reactive oxygen species
SIRT3	Sirtuin 3
SDH	Succinate dehydrogenase
SDHA	Succinate dehydrogenase subunit A
SDHAF1	Succinate dehydrogenase complex assembly factor 1
SDHAP1	Succinate dehydrogenase complex flavoprotein subunit a pseudogene 1
SDHB	Succinate dehydrogenase subunit B
SDHC	Succinate dehydrogenase subunit C
SDHD	Succinate dehydrogenase subunit D
SGI-110	Guadecitabine
SKP2	S-phase kinase associated protein 2
sRNA	Small non-coding ribonucleic acid
SUCLG1	Succinate-CoA ligase GDP/ADP-forming subunit alpha
SUCLG2	Succinate-CoA ligase GDP-forming subunit beta
SUCLA2	Succinate-CoA ligase ADP-forming subunit beta
SUCNR1	Succinate receptor 1
T2D	Type 2 diabetes mellitus
TCA cycle	Tricarboxylic acid cycle
TMZ	Temozolomide
TRAP1	Tumor necrosis factor receptor associated protein 1
TTFA	Thenoyltrifluoroacetone
